# Supplementary material for: Irregular sleep habits, regional grey matter volumes, and psychological functioning in adolescents
Source: PLoS One. 2021 Feb 10;16(2):e0243720. doi: 10.1371/journal.pone.0243720 (PMC7875363; doi:10.1371/journal.pone.0243720)
Supplement: Attachment — Submitted filename: PONE-D-19-34596.docx [file pone.0243720.s001.docx]

**Title:** Sleep, regional grey matter volumes, and psychological functioning in adolescent

**Manuscript Number:** PONE-D-19-34596

This manuscript deals with an interesting topic and is well written. I expect that if this manuscript is being published as an article in the PLOS ONE, it will draw a lot of attention from psychiatrists.

Although this study has several limitations (e.g. the cross-sectional nature, the use of a self-report sleep scale instead of an objective measure for sleep), the authors addressed these limitations well. Also, the authors tried to minimize potential confounding factors such as developmental effect or gender effect through the use of statistical adjustment or subgroup analysis.

**Minor concerns**

1. Was there any interaction effect between gender and sleep-related variables or gender and psychological functioning scores? Please present it in the result section if there was any (Line 252-255).
2. Please provide explanations for acronyms when first presented in the text (e.g., line 115)
3. Please provide p values throughout the text.
4. Please add an explanation for the acronym SDQ as a footnote in the tables (table 1, 3 and 4).
